# SPECT and PET imaging of angiogenesis and arteriogenesis in pre-clinical models of myocardial ischemia and peripheral vascular disease

**DOI:** 10.1007/s00259-016-3480-8

**Published:** 2016-08-12

**Authors:** Geert Hendrikx, Stefan Vöö, Matthias Bauwens, Mark J. Post, Felix M. Mottaghy

**Affiliations:** 1 Department of Nuclear Medicine, Maastricht University Medical Centre (MUMC+), Postbox 5800, 6202 AZ Maastricht, The Netherlands; 2 Department of Physiology, Maastricht University, Maastricht, The Netherlands; 3 Department of Nuclear Medicine, University Hospital, RWTH Aachen University, Pauwelsstr. 31, Aachen, 52072 Germany; 4 Cardiovascular Research Institute Maastricht (CARIM), Maastricht University, Maastricht, The Netherlands; 5 School of Nutrition and Translational Research in Metabolism (NUTRIM), Maastricht University, Maastricht, The Netherlands

**Keywords:** Radiotracer imaging, Angiogenesis, Arteriogenesis, Myocardial infarction, Peripheral vascular disease

## Abstract

**Purpose:**

The extent of neovascularization determines the clinical outcome of coronary artery disease and other occlusive cardiovascular disorders. Monitoring of neovascularization is therefore highly important. This review article will elaborately discuss preclinical studies aimed at validating new nuclear angiogenesis and arteriogenesis tracers. Additionally, we will briefly address possible obstacles that should be considered when designing an arteriogenesis radiotracer.

**Methods:**

A structured medline search was the base of this review, which gives an overview on different radiopharmaceuticals that have been evaluated in preclinical models.

**Results:**

Neovascularization is a collective term used to indicate different processes such as angiogenesis and arteriogenesis. However, while it is assumed that sensitive detection through nuclear imaging will facilitate translation of successful therapeutic interventions in preclinical models to the bedside, we still lack specific tracers for neovascularization imaging. Most nuclear imaging research to date has focused on angiogenesis, leaving nuclear arteriogenesis imaging largely overlooked.

**Conclusion:**

Although angiogenesis is the process which is best understood, there is no scarcity in theoretical targets for arteriogenesis imaging.

## Introduction

Molecular imaging enables the study of molecular and cellular processes in vivo [1]. Within this field, several noninvasive imaging techniques such as Magnetic Resonance imaging (MRI), Computed Tomography (CT), Optical Imaging (OI), Positron Emission Tomography (PET) and Single Photon Emission Computed Tomography (SPECT) are distinguished. The latter two are the most established techniques for targeting ongoing biochemical processes and are based on the detection of injected radiolabeled probes. While the spatial resolution of MRI and CT is higher, the detection sensitivity of PET and SPECT is within the picomolar or nanomolar range and therefore significantly higher than for MRI and CT [2, 3]. Spatial resolution and detection sensitivity are two performance characteristics that play an important role in molecular imaging research using SPECT and PET tracers. Clinical gamma cameras can provide a tomographic resolution of about 10 mm while preclinical devices currently reach submillimeter resolutions using a specialized multipinhole geometry [2, 4]. The difference between clinical and preclinical PET devices is smaller. While preclinical scanners reach spatial resolutions of 1–2 mm, clinical scanners operate within the range of 4–6 mm. The application for dedicated small animal SPECT and PET imaging modalities in preclinical models is highly valuable, as it has a great scope for noninvasive studying of dynamic biological processes at the molecular and cellular level [2]. Because of the high societal burden of disease, the cardiovascular system is a well-recognized target for molecular imaging. Longitudinal studies, monitoring cardiac function [5], imaging of atherosclerosis [6, 7], tissue viability and perfusion [8] and neovascularization [9, 10] are among the most studied cardiovascular areas. Molecular imaging of neovascularization has received a significant amount of attention as we still lack sensitive detection of neovascularization. It is assumed that such sensitive detection will facilitate translation of successful therapeutic interventions in preclinical models to the bedside [8, 11]. Neovascularization can be divided in three distinct processes, vasculogenesis, arteriogenesis and angiogenesis [12], and its extent determines the clinical outcome of coronary artery disease and other occlusive cardiovascular disorders. Vasculogenesis refers to the in situ formation of blood vessels from circulating endothelial progenitor cells. Despite the importance of this process during embryogenesis, its further discussion is beyond the scope of this review. The term arteriogenesis describes the enlargement of pre-existing arteriolar anastomoses into large collaterals in response to enhanced fluid shear stress [13]. Angiogenesis is an ischemia driven process that represents the sprouting of new capillaries from existing microvasculature [9].

Arteriogenesis is the most important mechanism in the functional replacement of an occluded artery in peripheral vascular disease (PVD) [13, 14], but the enlargement of coronary collateral arteries in obstructive coronary artery disease is also well described [15]. Angiogenesis is associated with postinfarct remodeling and has important implications for the prognosis following myocardial infarction (MI) [16], whereas its role in perfusion recovery in PVD is of less importance [13]. In this review, we will focus on SPECT- and PET-based neovascularization studies in the context of MI and peripheral vascular disease (PVD). As will become apparent from this review, extensive research has been conducted concerning radiotracer imaging of angiogenesis, while arteriogenesis radiotracer imaging is scarce and largely overlooked. Despite large parts of the pathways involved in arteriogenesis being unraveled, radiotracers specifically targeting this multifactorial process are yet to be developed. Alluding to the inferior amount of work being published on radiotracer imaging of arteriogenesis, we will briefly discuss the possible hurdles which have to be overcome in order to develop a nuclear arteriogenesis tracer.

## Perfusion tracers in neovascularization research

Although perfusion radiotracers do not directly target angiogenesis or arteriogenesis, they are used as indicators for areas of (mainly myocardial) ischemia, thereby often serving as a contrast in radiotracer-guided neovascularization research. The distribution kinetics of these tracers are therefore highly important for imaging of neovascularization. Perfusion tracers are even used as surrogate markers for neovascularization in pre-clinical research (“Radiotracer imaging of arteriogenesis” section). Accordingly, this section serves as a brief introduction into the uptake mechanisms, kinetics, and application of the most common SPECT and PET perfusion tracers.

Frequently employed perfusion tracers for SPECT are Thallium-201 (^201^Tl), Technetium-99 m (^99m^Tc)-sestamibi, ^99m^Tc-tetrofosmin and ^99m^Tc-pyrophosphate, while for PET, Oxygen-15 (^15^O)-water, N-13 (^13^N)-ammonia, Rubidium-82 (^82^Rb) and the more recently developed Fluorine-18 (^18^F)-labeled Flurpiridaz (Lantheus Medical Imaging, Massachusetts, USA) are the most common perfusion tracers.

^201^Tl is taken up in viable cells via the sodium-potassium pump as it has properties similar to potassium [17]. However, while ^201^Tl has successfully been used in cardiac perfusion imaging [18] and in skeletal muscle perfusion imaging in PVD patients [19–22], ^99m^Tc-labeled perfusion tracers have largely replaced the use of ^201^Tl. Beside the considerably lower radiation exposure (6 vs. 28 millisievert) ^99m^Tc-labeled tracers offer more advantages compared to ^201^Tl, the most essential being the shorter half-life (6 h for ^99m^Tc compared to 73 h for ^201^Tl), allowing for injection of higher doses, in combination with the higher energy level at which ^99m^Tc emits gamma rays [140 k electronvolt (keV) compared to 78 keV for ^201^Tl], which results in less scatter and attenuation. Together, these advantages culminate in improved imaging [23].

One ^99m^Tc-labeled compound in particular, ^99m^Tc-sestamibi, is omnipresent in clinical cardiology [24] and has also been incorporated in several studies examining lower-extremity perfusion in PVD [25–27]. ^99m^Tc-sestamibi is a lipophilic, cationic complex of six isonitriles [23]. Like ^201^Tl, uptake of ^99m^Tc-sestamibi after intravenous injection is proportional to blood flow [28]. Cellular uptake and retention of ^99m^Tc-sestamibi are dependent on mitochondrial and plasma membrane potentials [29–31]. After uptake, the compound resides in myocardial cells after initial extraction and demonstrates minimal delayed redistribution [32–34]. In a case report, the merit of clinical application of ^99m^Tc-sestamibi over Doppler ultrasound in PVD patients has already been reported on the basis of improved sensitivity in detecting differences in detecting differences of resting perfusion between the lower extremities [35]. ^99m^Tc-tetrofosmin is an alternative lipophilic cationic complex with comparable uptake characteristics and similar widespread use in myocardial perfusion imaging [24]. However, the hepatobiliary clearance of ^99m^Tc-tetrofosmin is reported to be slightly faster than for ^99m^Tc-sestamibi [23]. Recently, Stacy et al. showed preliminary data and indicated that SPECT/CT using ^99m^Tc-tetrofosmin has the potential to assess regional differences in lower-extremity perfusion in PVD patients. Furthermore, ^99m^Tc-pyrophosphate, binding to hydroxyapatite crystals in damaged myocytes, has been frequently employed in clinical practice to identify fresh myocardial infarctions since its introduction in 1974 [36, 37]. Additionally, ^99m^Tc-pyrophosphate has been successfully used to estimate the ischemic skeletal muscle mass in a canine ischemia-reperfusion skeletal muscle model [38] and in PVD patients [39].

The most prominent PET perfusion tracers are ^15^O-water and ^13^N-ammonia. Both tracers have a short half-life (2.4 min and 9.8 min respectively) requiring an on-site cyclotron to enable application, thereby limiting the use of these tracers to a few centers [40]. Myocardial blood flow acquired with ^15^O-water and ^13^N-ammonia have been widely validated against independent microsphere blood flow measurements in animals and have yielded highly reproducible values over a range of 0.5 to 5.0 ml/g/min. [40–42] ^15^O-water, diffusing freely into the tissue, is also frequently implemented in PVD patient studies [43–47]. The characteristics of ^15^O-water make the tracer suitable for repeated measurements during a single visit, measurements at rest and during exercise or during vasodilator stress. An ^15^O-water rest-stress PET study found significantly lower calf muscle flow reserve in PVD patients compared to healthy control subjects, and these measurements correlated with thermodilution-derived flow reserve values [45]. Furthermore, a study by Scremin et al. showed that accurate muscle blood flow detection by ^15^O PET in legs with severe ischemia could add valuable information about skeletal muscle viability in the residual limb when deciding the level of an amputation [46]. However, despite its frequent application, ^15^O-water images of the myocardium are commonly of lower count density due to subtraction of the blood pool, rapid clearance of ^15^O-water and its short half-life. Therefore, ^15^O-water images are not suitable for the visual analysis of myocardial radiotracer uptake, and thus are not used clinically for coronary artery disease detection [40, 48]. ^13^N-ammonia is cleared rapidly from the circulation and is primarily taken up by the myocardium, brain, liver, kidneys and the pituitary gland [49, 50]. In both myocardium and brain, ^13^N-ammonia is removed from the blood by first-pass extraction (approximately 80 %) and is metabolically trapped within the tissues by incorporation into the cellular pool of amino acids, mainly as glutamine [50–52]. The high first pass extraction, in combination with a sufficiently long half-life, allow high count images to be acquired. Hence, flow-limiting coronary artery disease can be visualized on stress-rest images using ^13^N-ammonia [53, 54]. While ^13^N-ammonia PET is frequently used to measure myocardial perfusion, its application for measuring skeletal muscle perfusion is rare, though not absent. In a patient with a right-sided static tremor, higher uptake of ^13^N-ammonia was found in the muscles of the right leg, which was related to increased perfusion produced by continuous exercise of the muscles involved in the tremor [55]. Furthermore, ^13^N-ammonia PET was successfully used to measure local perfusion in the legs of patients with painful diabetic neuropathy [56].

^82^Rb, a functional potassium analog, is an alternative radioactive tracer of myocardial perfusion that can be imaged with PET [24, 40]. Its diagnostic and prognostic performances appear comparable to conventional blood flow SPECT imaging [57, 58]. Although ^82^Rb can be eluted from a commercially available Strontium-82 generator on site [40], a major limitation is its ultrashort half-life (76 s), which limits its use to pharmacological stress perfusion imaging [24].

A promising ^18^F-labeled perfusion tracer was added to the available PET perfusion tracers almost a decade ago in the form of Flurpiridaz (initially evaluated as: BMS-747158-02; Lantheus Medical Imaging, Massachussets USA). Flurpiridaz is an analog of the insecticide pyridine, which binds to the mitochondrial complex I of the electron transport chain with a very high affinity [40, 59, 60]. The radiotracer is rapidly cleared from the blood (in under 5 min) and displays stable uptake in the healthy and infarcted myocardium up to 40 min. Furthermore, 18F-Flurpiridaz has a high first-pass extraction fraction above 90 % (which is preserved at high flow rates) and a very slow wash out [40]. These favorable properties in combination with its half-life of 109 min result in high count images of high diagnostic quality for the detection of perfusion deficits underlying coronary artery disease (CAD) [40, 61, 62]. ^18^F-Flurpiridaz myocardial blood flow PET imaging was validated using radioactive microspheres in a pig model [40, 63]. Moreover, positive results from phase 2 human studies have been published [61]. The high extraction fraction of ^18^F-Flurpiridaz may offer an advantage for evaluating lower-extremity skeletal muscle blood flow. However, so far there are no studies that assessed the potential of ^18^F-Flurpiridaz in the setting of PVD.

## Radiotracer imaging of angiogenesis

### Angiogenesis

**Fig. 1:**
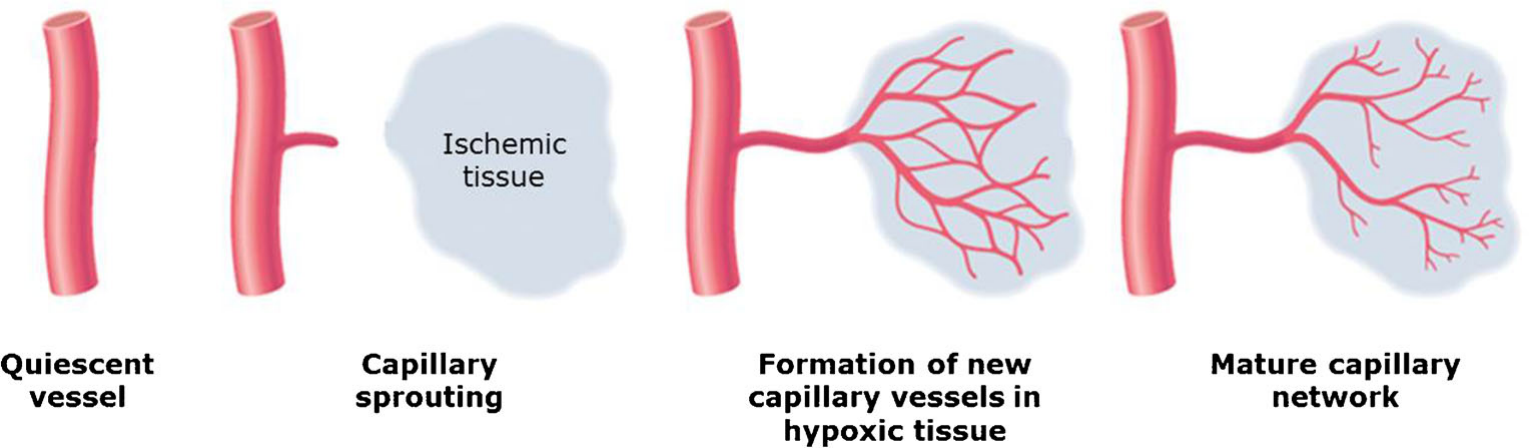
Mechanism of angiogenesis. Capillary sprouting is guided into the ischemic area down a chemotactic gradient of growth factors. Modified from Carmeliet, 2000, Nature Medicine [12]

The formation of new capillary arteries from pre-existing microvasculature is termed angiogenesis. Angiogenesis is a dynamic process involving endothelial proliferation and differentiation which is mainly triggered by tissue ischemia or hypoxia. During this process, new capillaries form around ischemic tissue zones, as they occur in MI, stroke, and PVD [64, 65]. Upon development of tissue ischemia, transcription factors such as hypoxia inducible factor 1α (HIF-1α) and inflammatory mediators are released locally resulting in vasodilation, enhanced vascular permeability, and accumulation of monocytes and macrophages, which in turn secrete more growth factors and inflammatory mediators [65, 66]. These inflammatory cells facilitate degradation of the basal membrane of the parent artery and the surrounding extracellular matrix (ECM) through the release of matrix metalloproteinases (MMPs). Following ECM degradation, endothelial cells migrate and proliferate down a hypoxia-sensitized chemotactic gradient of various growth factors to form a new capillary vessel with a lumen [65]. The role of integrins in this part of the angiogenic process is of paramount importance, as integrins are the principle adhesion receptors used by endothelial cells to interact with their extracellular microenvironment [67]. The subsequent formation of a functioning vasculature requires the orchestrated interaction of endothelial cells, the extracellular matrix, and surrounding cells such as pericytes and smooth muscle cells [65, 68]. This sprouting process iterates until proangiogenic signals abate, and quiescence is re-established [69] (Fig. 1).

Angiogenesis is a multistep process orchestrated by a multitude of angiogenic factors and inhibitors, which offer a wide range of targets for therapeutic interventions and imaging [9, 68]. Because of its important role in the (partial) restoration of tissue perfusion in the ischemic area, angiogenesis stimulating therapy is an intensely studied subject in cardiovascular research. Nevertheless, while results from animal studies have been encouraging [70–74], the results obtained during clinical studies have not been convincing [75–78]. Plausible explanations for the latter are ineffective growth factor delivery, irreproducible readout parameters, and an unresponsive patient population [79]. However, the most important feature in a therapeutic intervention study is its ability to accurately monitor the targeted process. Current clinical readout parameters such as peak walking distance in PVD trials [80] and the exercise tolerance test in coronary artery disease trials [78] are not sufficiently sensitive and reproducible. Surely, perfusion imaging through MRI, SPECT and PET imaging can be used to indicate enhanced perfusion of the ischemic tissue. However, it often takes several months before improvement becomes apparent [8]. In order to facilitate early diagnosis and early treatment for patients with ischemic cardiovascular disease specific and sensitive non-invasive tracers for neovascularization will be required.

### Main targets for targeted radiotracer imaging of angiogenesis in myocardial ischemia

The main targets for nuclear angiogenesis imaging in animal models of MI are the α_v_β_3_ integrin and the vascular endothelial growth factor (VEGF)-receptor, while CD105 (endoglin) and CD13 (aminopeptidase-N) were also successfully used (Table 1).

**Table 1:** Radiotracers for myocardial angiogenesis imaging in pre-clinical studies

Myocardial angiogenesis			
Biological target	Tracer	Modality	Reference(s)
A_v_β_3_ integrin	^99m^Tc-NC100692	SPECT	[81–83]
	^99m^Tc-RAFT-RGD	SPECT	[84]
	^111^In-RP748	SPECT	[16, 85, 86]
	^123^I-gluco-RGD	SPECT	[87]
	^18^F-galacto-RGD	PET	[88, 89]
	^18^F-AlF-NOTA-PRGD2	PET	[90]
	^68^Ga-NOTA-RGD	PET	[91, 92]
	^68^Ga-NODAGA-RGD	PET	[89]
	^68^Ga-TRAP(RGD)_3_	PET	[89]
CD13	^111^In-DTPA-cNGR	SPECT	[8]
VEGF receptor	^64^Cu-DOTA-VEGF_121_	PET	[93]
CD105	^64^Cu-NOTA-TRC105	PET	[94]

Integrins are transmembrane receptors that contribute to the angiogenic process through increased signal transduction as well as modulation of cell adhesion to the extracellular matrix [47, 67, 95]. The α_v_β_3_ integrin (also termed vitronectin receptor) is the most abundant integrin expressed on the surface of proliferating endothelial cells and has been implicated in cell migration and cell survival signaling. Further, the α_v_β_3_ integrin is minimally expressed on normal quiescent endothelial cells [96]. These properties have made the α_v_β_3_ integrin a target of choice for the imaging of angiogenesis. The arginine-glycine-aspartic acid (RGD) peptide, naturally present in extracellular matrix proteins, was found to be highly selective for the α_v_β_3_ integrin. The discovery of the RGD sequence has marked the starting point for the development of probes targeting the α_v_β_3_ integrin [9, 96]. RGD peptides evolved from linear peptides with low selectivity and biostability to constructs with better pharmacokinetics (through attachment of carbohydrates, charged amino acids and polyethylene glycol groups) and optimized binding characteristics (through multimerization) [9, 96].

Currently, a large variety of RGD peptides have been developed that are suited for radiolabeling into SPECT or PET tracers for non-invasive imaging of angiogenesis. Pre-clinically, these tracers have been tested in animal models for MI, with and without reperfusion and in the presence of pro-angiogenic growth factors, and in hind limb ischemia. Enhanced angiogenesis post-MI has been indicated by several groups using Technetium-99 m (^99m^Tc)-labeled RGD peptides. ^99m^Tc-NC100692 (maraciclatide®) is a technetium-labeled cyclic RGD peptide that has been used in a variety of SPECT studies to non-invasively assess angiogenesis [97]. This compound has a high affinity for the α_v_β_3_ integrin, is metabolically stable, and has a biodistribution and kinetics that are favorable for SPECT imaging. SPECT imaging of ^99m^Tc-NC100692 was successfully used to indicate post-MI angiogenesis hypoperfused (indicated by ^201^Tl) myocardial regions of matrix-metalloproteinase-9 (MMP-9) null mice compared to wild-type mice [83]. In rats, SPECT imaging of ^99m^Tc-NC100692 was used to indicate ongoing angiogenesis in the peri-infarct region after MI [81] and in reperfused ischemic myocardium [82]. Furthermore, ^99m^Tc-RAFT-RGD, a different RGD-based tracer for targeting α_v_β_3_ integrin expression in vivo, was used in a rat model of reperfused ischemic myocardium. SPECT imaging was used to show enhanced ^99m^Tc-RAFT-RGD, but not ^99m^Tc-RAFT-RAD (negative control), uptake in the infarct and peri-infarct zone 14 days after reperfusion [84]. Additionally, specific binding of ^99m^Tc-RAFT-RGD to the α_v_β_3_ integrin was shown in human microvascular endothelial cells, as the presence of an excess of unlabeled RGD resulted in a significant inhibition of ^99m^Tc-RAFT-RGD binding.

The suitability of the α_v_β_3_ integrin as a target for radiotracer imaging of angiogenesis was further established by studies that employed the indium-111 (^111^In)-labeled RP748 (also named quinolone). ^111^In-RP748 is an α_v_β_3_ integrin binding small molecule that specifically binds to activated endothelial cells in vitro and vivo [98]. This SPECT radiotracer has subsequently been assessed in rat [16, 85, 86] and canine [16, 86] models of MI in both early (acute) and late (3 weeks) time points after induction of MI. Binding specificity of ^111^In-RP748 was shown by a direct comparison with the control compound ^111^In-RP790 having similar chemical structure, although no in vitro specificity for α_v_β_3_-integrin. SPECT imaging revealed no uptake of ^111^In-RP790 in hypoxic or infarct areas [16, 86].

Furthermore, in a swine model of hibernating myocardium, SPECT imaging revealed enhanced Iodine-123 (^123^I)-gluco-RGD uptake in areas corresponding to ^201^Tl defects in animals that received an endomyocardial injection of VEGF, compared to animals that received a saline control injection. No uptake of an ^123^I-labeled control peptide was seen in the heart of a control animal [87].

Despite the variety in SPECT radiotracers for angiogenesis, PET radiotracers for angiogenesis have actually been investigated more frequently, with results that are very similar to studies that use SPECT imaging in preclinical models of cardiac angiogenesis. Tracers that have been used include ^18^F-galacto-RGD [88, 89], ^18^F-AlF-NOTA-PRGD2 [90], ^68^Ga-NOTA-RGD [91, 92], ^68^Ga-NODAGA-RGD [89], and ^68^Ga-TRAP(RGD)_3_ [89]. Remarkably, in some studies, the uptake of RGD-based PET tracers was enhanced up to 4 [90] or even 6 [88] months after the angiogenesis stimulating intervention. Specificity of uptake was shown either by inhibition of binding with a specific non-radiolabeled α_v_β_3_ integrin antagonist [88] or by co-incident and co-localized endothelial integrin markers such as CD31 or CD61 (β_3_) [89, 90, 92].

Together with the α_v_β_3_ integrin, the RGD peptide forms a reliable axis for targeted radiotracer imaging of angiogenesis. However, despite the promising results obtained in pre-clinical studies, the application of RGD-based radiotracers for angiogenesis imaging in the context of myocardial ischemia or PVD in the clinic is modest to say the least. Instead radiotracer imaging of angiogenesis in patients so far has largely focused on imaging of tumor angiogenesis. As in pre-clinical research, the main target for imaging has been the α_v_β_3_ integrin through various radiolabeled RGD peptides [99–106], while the VEGF receptor [107], prostate-specific membrane antigen (PSMA) [108], and the extra domain B of fibronectin [109] have also received attention. To date, only a few small studies in MI patients have been performed, all using the α_v_β_3_ integrin as a target for angiogenesis imaging [110–112] (Table 2).

**Table 2:** Radiotracers for myocardial angiogenesis imaging in clinical studies

Myocardial angiogenesis			
Biological target	Tracer	Modality	Reference
A_v_β_3_ integrin	^18^F-galacto-RGD	SPECT	[110]
	^99m^Tc-NC100692	PET	[111]
	^68^Ga-PRGD2	PET	[112]

Makowski et al. used PET/CT imaging to target the α_v_β_3_ integrin with ^18^F-galacto-RGD in an MI patient, and found enhanced uptake in infarcted area (defined by the extent of delayed enhancement MRI and decreased ^13^N-ammonia myocardial blood flow) 2 weeks after MI. The feasibility of clinical angiogenesis imaging through targeting the α_v_β_3_ integrin was further shown in studies by Mozid et al. [111] and Sun et al. [112]. Mozid and co-workers applied an intracoronary injection of granulocyte colony-stimulating factor mobilized bone-marrow stem cells in patients with chronic ischemic heart failure. Using ^99m^Tc-NC100692 SPECT imaging they found baseline (day 0) uptake in all heart failure patients with no uptake seen in control patients. This suggests persistent angiogenesis in patients with chronic heart failure and remote MI, which is in line with the preclinical finding of enhanced uptake long after the ischemic incident. Unfortunately, no proof of concept was provided that therapy-induced neovascularization can be picked up by RGD-based PET imaging in a robust manner [111]. Sun and co-workers used ^68^Ga-PRGD2 SPECT in MI and stroke patients and found enhanced uptake in 20 out of 23 MI patients and in eight out of 16 stroke patients. Furthermore, higher uptake of ^68^Ga-PRGD2 was observed 1–3 weeks after the onset of MI/stroke and correlated well with the disease phase and severity [112].

Large volume patient studies employing RGD-based radiotracers for imaging of post-MI angiogenesis imaging are currently lacking, despite the fact that there is no scarcity in tracer constructs. This begs the question if this is because of uncertainty if targeted angiogenesis imaging has clinical benefit due to unconvincing results in the small scale clinical studies, or because there is lack of scientific interest in the absence of approved therapeutic angiogenesis? The amount of pre-clinical studies suggests there is no lack of scientific interest, so it is assumed that the field is held up by a lack of therapeutic programs.

Fortuitously, angiogenesis is hallmarked by the upregulation of multiple biomarkers. Beside upregulation of the α_v_β_3_ integrin, CD13, a membrane bound aminopeptidase, is also upregulated on angiogenically active endothelial cells [113, 114]. CD13, expressed on active endothelial cells, can specifically be targeted using an asparagine-glycine-arginine (NGR) peptide motif. [114] Competition studies in tumor angiogenesis with the NGR and RGD motifs demonstrated a threefold higher target homing ratio (tumor/control organ) for NGR than for RGD [115]. In a recent study by our own group, CD13 was targeted with a cyclic asparagine-glycine-arginine (NGR) peptide, having a tenfold higher targeting efficacy than the linear entity [116], which was coupled to ^111^In via a diethylene triamine pentaacetic acid (DTPA) chelator. Dual isotope SPECT imaging indicated significantly enhanced uptake of ^111^In-DTPA-cNGR mainly in areas of ^99m^Tc-sestamibi absence (infarct region) [8]. Given the higher target homing ratio compared to the RGD motif, it is interesting to speculate that CD13 targeting through NGR-based tracers could lead to better image quality and subsequently better possibilities for clinical translation. However, studies comparing NGR and RGD-based tracers in the same model have to be conducted before such claims can be justified. Furthermore, to gain insight into the true benefit of monitoring angiogenesis in comparison with traditional endpoints or indirect effects such as clinical state, or tissue perfusion or function, large volume patient studies have to be conducted.

#### Other targets for radiotracer imaging of angiogenesis in myocardial ischemia

Although various angiogenesis stimulating factors exist, VEGF is considered the most potent and predominant factor [79, 95]. VEGF ligands, of which there are four known isoforms (A-D), are released in response to ischemia and mediate their angiogenic effects by binding to specific VEGF receptors (VEGFR-1, VEGFR-2 and VEGFR-3), leading to receptor dimerization and subsequent intracellular signal transduction via tyrosine kinases [117, 118]. The majority of VEGF-based radiotracers have been evaluated in the context of tumor angiogenesis imaging. However, peripheral angiogenesis and post MI angiogenesis have been examined as well.

In a rat MI model, increased myocardial uptake in infarcted myocardium (visualized by ^18^F-FDG) of the PET radiotracer Copper-64 (^64^Cu)-DOTA-VEGF_121_ was shown on day 3, 7 and 17 after induction of MI. Myocardial origin of the radiotracer signal was confirmed by CT co-registration and autoradiography [93].

Among the targets that received less attention while being successfully used for nuclear imaging of angiogenesis are CD105, a 180 kDa disulfide-linked homodimeric transmembrane protein selectively expressed on the endothelial cells of newly formed vessels [119–122], and CD13, a membrane bound aminopeptidase found on activated endothelial cells [113]. PET imaging of CD105 expression in a rat MI model with ^64^Cu-labeled TRC105, an anti-CD105 monoclonal antibody, revealed significantly enhanced uptake in infarcted myocardium (indicated by ^18^F-FDG) 3 days after surgically induced MI compared to sham operated control animals. These findings were supported by histology, indicating increased CD105 expression following MI [94].

### Radiotracer imaging of angiogenesis in peripheral vascular disease

PVD is a progressive atherosclerotic process that results in stenosis or occlusion of non-coronary blood vessels, most frequently the iliac and femoral artery [47]. Progressive ischemia, present in PVD, can lead to intermittent claudication, non-healing ulcers, limb amputation and in severe cases, death [47, 123]. Despite the severity of this disease, a significant proportion of individuals with PVD remain undiagnosed in clinical practice [124].

Although angiogenesis might have less impact on perfusion recovery in PVD than arteriogenesis, targeted nuclear imaging of angiogenesis can provide valuable information of the underlying pathophysiology associated with PVD. Especially in combination with lower-extremity perfusion imaging, areas of ischemia can be identified that might have remained unnoticed on MRI images or other techniques used in present day clinical care (e.g. ankle-brachial index, duplex ultrasound or CT angiography) [47].

In pre-clinical research, numerous hind-limb ischemia models have been established in several laboratory animals to mimic the situation of PVD and to study (stimulation of) neovascularization [125]. Like in MI models, targeted imaging of angiogenesis in pre-clinical models of PVD has mainly focused on the α_v_β_3_ integrin and VEGF receptors (Table 3).

**Table 3:** Radiotracers for peripheral angiogenesis imaging in pre-clinical studies

Peripheral angiogenesis			
Biological target	Tracer	Modality	Reference(s)
A_v_β_3_ integrin	^99m^Tc-NC100692	SPECT	[126, 127]
	^68^Ga-NOTA-RGD	PET	[128]
	^125^I-c(RGD(I)yV)	SPECT	[129]
	^76^Br-Nanoprobe	PET	[130]
VEGF receptor	^111^In-VEGF_121_	SPECT	[131]
	^64^Cu-VEGF_121_	PET	[132]
NPR-C	^64^Cu-DOTA-CANF-comb	PET	[133]
CD105	^64^Cu-NOTA-TRC105	PET	[134]

#### The α_v_β_3_ integrin

The majority of RGD-based radiotracers that have beenh developed have been tested in cardiac angiogenesis models. However, among them, ^99m^Tc-NC100692 [126, 127] and ^68^Ga-NOTA-RGD [128] have also been evaluated in preclinical PVD models. Additionally, ^125^I-c(RGD(I)yV) [129] and a bromine-76-labeled nanoprobe (^76^Br-Nanoprobe) [130] have only been tested in a mouse model for PVD. Specific radiotracer uptake was concluded from the absence of uptake of a scrambled control peptide [126, 129], inhibition of binding using an excess of the non-radiolabeled tracer [128, 130] or by co-localized binding of a fluorescent tracer analogue and CD31 [127]. With the majority of studies only assessing radiotracer uptake at relatively short time points after induction of ischemia (i.e. up to 14 days) [127–130] and only one study assessing uptake after 4 weeks [126], the possibility and benefit of radiotracer guided imaging of neovascularization in PVD models at later time points remains to be discovered.

#### Growth factor receptors

The involvement of numerous growth factors such as VEGF and FGF in neovascularization has been described, and also in PVD [135]. Growth factor functions are regulated in a complex fashion with multiple feedback systems influencing many cell types; hence, it is extremely difficult to elucidate unique roles of each growth factor unless it is specific to a single cell type [136]. Therefore, rather than targeting the growth factor itself for molecular imaging, their most important or abundant receptors have been used as targets, as these are thought to be more specifically regulated during neovascularization than their ligands. For example, the extracellular matrix (ECM) serves as a reservoir for growth factors [137], thereby forming a storage that can be tapped on demand. Detecting the presence of growth factors by molecular imaging could therefore be unrelated to active neovascularization.

As VEGF is one of the dominant growth factors inducing angiogenesis, its functional receptor, VEGFR2 has been targeted frequently for molecular imaging. Using VEGF_121_, a natural splice variant of VEGF_165_ that lacks an ECM reservoir binding capacity [138], the VEGFR2 receptor can be visualized in relationship to neovascularization events. In a rabbit hind limb ischemia model, 10 days after arterial ligation, uptake of ^111^In-VEGF_121_ was enhanced as shown by SPECT imaging and post-mortem gamma counting [131]. Corresponding immunohistological findings of increased VEGFR2 expression validated the principle. A similar finding was reported using PET and ^64^Cu-labeled VEGF_121_ in a mouse hindlimb ischemia study, where the level of uptake correlated well with VEGFR2 protein levels in ligated and control limbs in the presence and absence of exercise [132].

#### Other targets for radiotracer imaging of angiogenesis in peripheral vascular disease

Other targets that were successfully used for nuclear imaging of peripheral angiogenesis are CD105 and the natriuretic peptide clearance receptor (NPR-C). Among the four natriuretic peptide family members, all binding the NPR-C, atrial natriuretic peptide and C-type natriuretic peptide have been demonstrated to suppress VEGF signaling and to attenuate angiogenesis [139–142]. Liu et al. developed a C-type atrial natriuretic factor (CANF)-conjugated comblike nanoprobe that was labeled with ^64^Cu. In a mouse model of hind limb ischemia PET imaging of ^64^Cu-DOTA-CANF-comb showed a significantly higher uptake in the ischemic hind limb compared to the nonischemic control limb 7 days after induction of ischemia. These results were supported by immunohistochemical findings of NPR-C upregulation with colocalization in endothelial (via PECAM-1 staining) and smooth muscle cells (via α-actin staining) [133].

Furthermore, the PET tracer ^64^Cu-NOTA-TRC105 was used to assess the response to pravastatin treatment in a mouse ischemic hind limb model. Pravastatin is a member of the statin group of cholesterol-lowering drugs that is also known to stimulate NO-mediated angiogenesis. Significantly increased radiotracer uptake in the ischemic hind limb compared to the control hind limb was shown at day 3, 10, 17 and 24 after induction of hind limb ischemia in the pravastatin treated group with CD31/CD105 co-immunostaining validating the radiotracer uptake [134].

In sharp contrast to targeted neovascularization imaging in cancer patients and to a lesser extent in MI patients, radiotracer imaging in PVD patients is based entirely on the use of perfusion tracers rather than on targeted imaging of neovascularization. Although specific angiogenesis tracers are readily available, it would be more appropriate and helpful to monitor arteriogenesis, as it is by far the most efficient adaptive mechanism of survival for ischemic limbs [143]. However, in the absence of specific arteriogenesis tracers, perfusion tracers are currently used for diagnosis and monitoring of perfusion recovery. In order to improve diagnosis and therapy monitoring in PVD, but also in MI patients, the development of specific arteriogenesis tracers is warranted.

## Radiotracer imaging of arteriogenesis

### Arteriogenesis

Since the first observations by Fulton in 1964 [144], our knowledge about arteriogenesis and its underlying cellular and molecular mechanisms has increased vastly, though the fundamental event that initiates mitogenic stimulation has not been unraveled as of today.

**Fig. 2:**
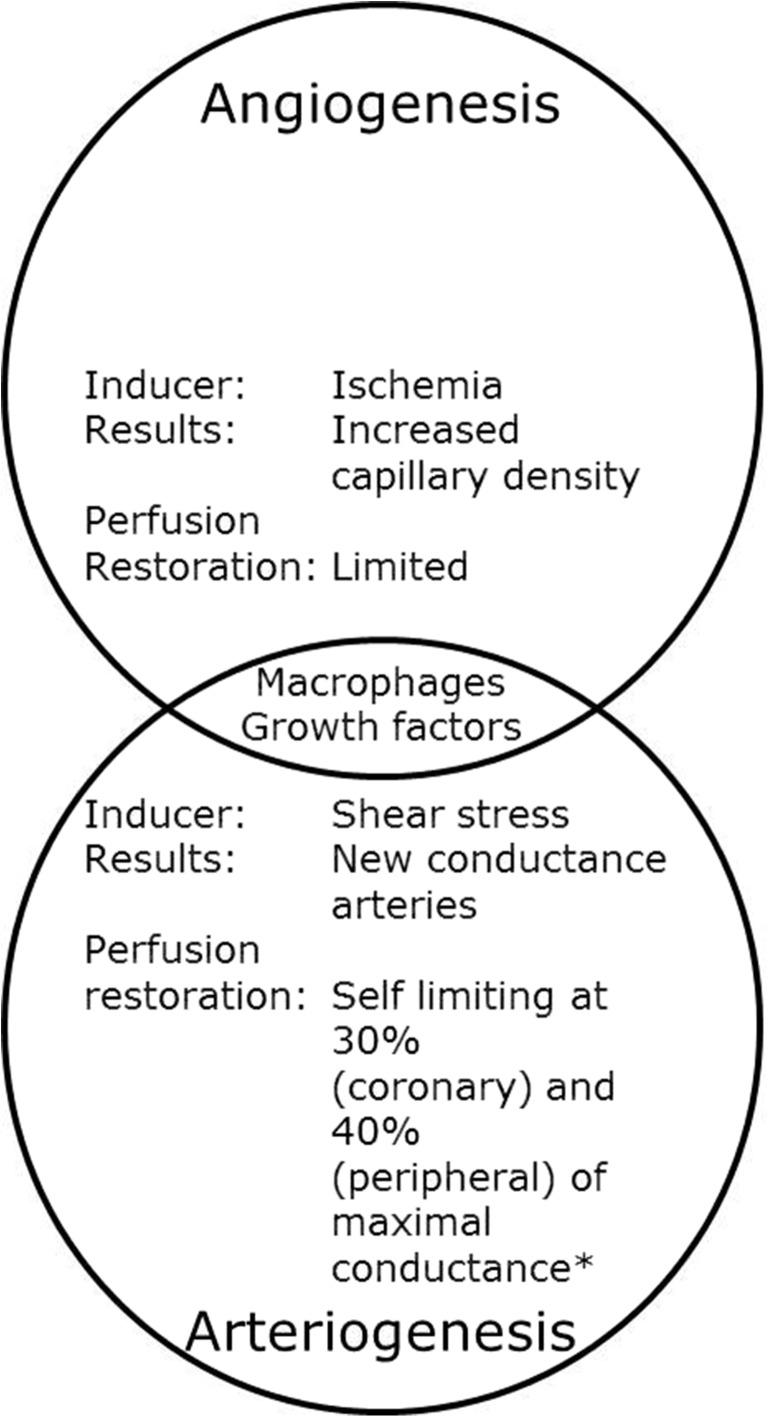
Key features in angiogenic and arteriogenic vessel growth. Both processes share their dependency on macrophage guided, controlled extracellular matrix and vessel scaffold degradation. Nevertheless, both the initial stimulus and the outcome differ significantly between both processes. *Established in pre-clinical models. Modified from Buschmann and Schaper, 1999, Physiology [145]

**Fig. 3:**
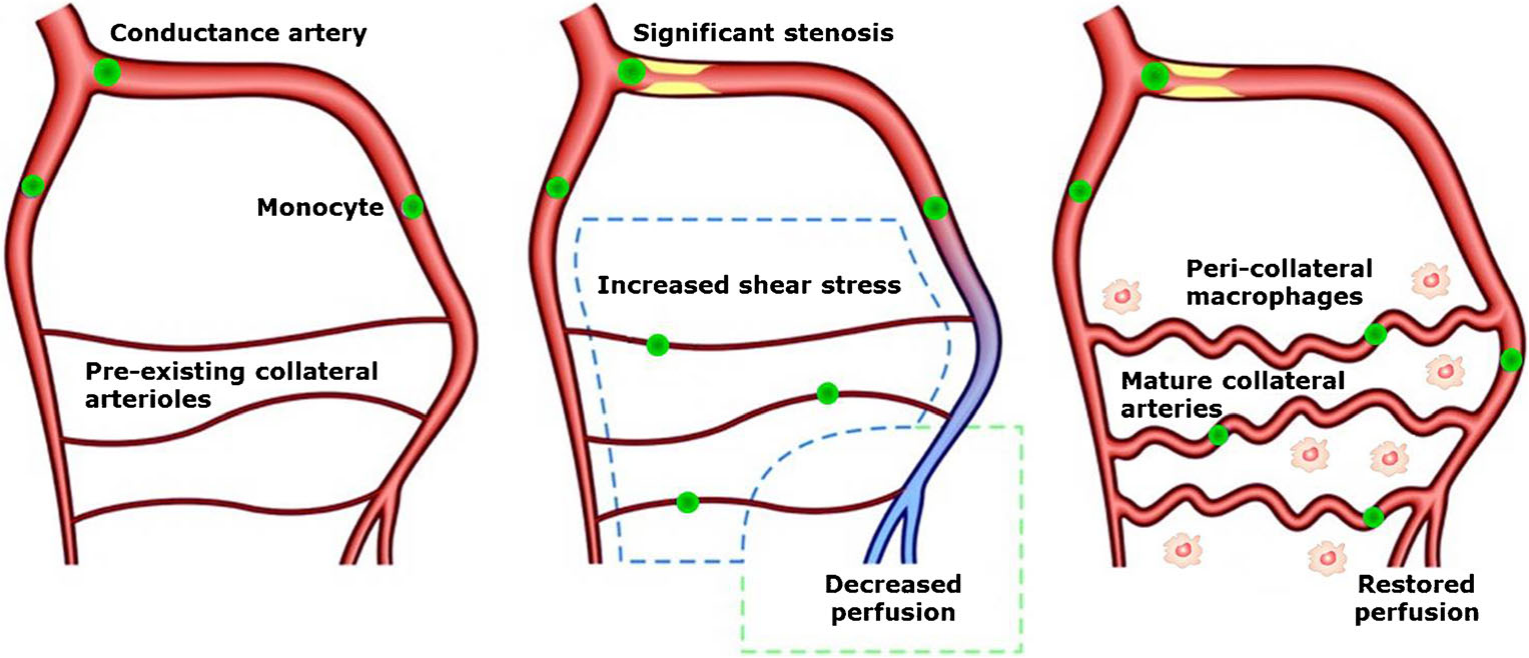
Mechanism of arteriogenesis. Increased shear stress over pre-existing collateral arterioles triggers a macrophage guided outward remodeling that results in the (partial) restoration of perfusion. Modified from Schirmer et al., 2009, Heart [146]

The initiation of arteriogenesis is, in sharp contrast to angiogenesis, independent of ischemia and instead relies on physical factors (Figs. 2 and 3). Following the occlusion of a conductance artery, it is generally accepted that the arteriogenic process is initiated in the pre-existing collaterals that circumvent the obstruction by deformation of the endothelial cells as a consequence of increased pulsatile fluid shear stress. Initially, these pre-existing collateral arteries are incapable of conducting the mandatory blood supply to the tissue that is situated distally of the occlusion. In order to cope with the increased perfusion pressure, diametrical growth and artery maturation of the pre-existing collateral artery are required. Successful maturation into a conductance artery relies on the creation of a transient inflammatory environment. Increased expression of adhesion molecules, cytokines and growth factors by the endothelium in response to increased shear stress hallmark the inaugural events in a complex cascade [147]. Subsequently, circulating monocytes attach to the endothelium, migrate to the peri-collateral space, and differentiate into macrophages [148, 149]. The inflammatory reaction that ensues is vital to arteriogenesis and driven by the secretion of growth factors and cytokines from endothelial cells, smooth muscle cells, monocytes, and macrophages. Among these secreted factors are monocyte chemotactic protein 1 (MCP1), which induces the attraction of more monocytes; tumor necrosis factor α (TNFα), which provides the inflammatory environment in which collateral vessels develop; and MMPs that control the digestion of the internal elastic lamina and the surrounding extracellular matrix [148]. Simultaneous to the controlled digestion of the extra-cellular scaffolding, a burst of mitotic activity of smooth muscle cells and endothelial cells is initiated, resulting in an outward remodeling and a subsequent larger cross sectional area of the collateral artery. The enhanced cross sectional diameter causes the blood flow velocity and shear stress to normalize. Hence, the arteriogenic process is self-limiting after collateral arteries reach a sufficiently large diameter. Further maturation of the vessel occurs through the orderly arrangement of smooth muscle cells in circular layers, establishment of cell-cell contacts and the synthesis of elastin and collagen [150].

However, despite the extensive pre-existing collateral network (and thereby possible opportunities for arteriogenic repair) in the human body, most molecular neovascularization imaging research to date has focused on angiogenesis, leaving molecular arteriogenesis imaging largely overlooked. This is not particularly useful, as arteriogenesis is more capable of restoring tissue blood supply than angiogenesis [15, 151], as collateral vessels have the capacity to carry a larger volume of blood than sprouting capillary networks [152]. At present, determining precise morphology is still dependent on post mortem angiography, as some collateral arteries (i.e. collateral arteries in the subendocardial plexus of the left ventricle) are rather poorly represented in clinical angiography [15]. Molecular tracers for arteriogenesis imaging are also lacking despite the identification of a fairly extensive set of molecular circuits that are perturbed [150].

### Radiotracer imaging of arteriogenesis in models for myocardial ischemia

Despite the lack of specific radiotracers for arteriogenesis, there are studies in which histological findings to indicate ongoing arteriogenesis were supported by nuclear imaging (and vice versa), both in the context of MI as well as PVD. As already alluded to in the “Perfusion tracers in neovascularization research” section in this review, perfusion tracers for PET and SPECT can be used for this purpose.

Li et al. employed gene therapy using a plasmid-encoding, human-platelet-derived endothelial cell growth factor (PD-ECGF) cDNA in a dog chronic myocardial ischemia model. A double immunohistochemical staining for von Willebrand factor and α smooth-muscle actin (αSMA) demonstrated that angiogenesis and arteriogenesis occurred. These findings were accompanied by enhanced myocardial blood flow 2 weeks after induction of gene therapy, as indicated by N^13^-ammonia PET imaging [153]. In a different study, Zuo et al. subjected pigs to coronary artery ligation and subsequent intramyocardial administration of a recombinant adeno-associated virus construct coding CD151 (a tetraspanin superfamily protein). Using N^13^-ammonia PET, they found significantly enhanced regional myocardial perfusion 8 weeks after viral transduction, which was accompanied by a marked increase in capillary (indicated by von Willebrand staining) and arteriolar density (indicated by αSMA staining) [154]. Furthermore, in rats subjected to coronary artery ligation, Kainuma et al. showed that combined treatment of the ischemic myocardium with skeletal myoblast cell-sheet plus an omentum-flap resulted in a greater amount of functionally (CD31+/Lectin+) and structurally (CD31+/αSMA+) mature blood vessels 4 weeks after treatment. Additionally, N^13^-ammonia PET showed better global coronary flow reserve in the group receiving this combined treatment [155].

While the majority of studies linking enhanced myocardial blood flow to enhanced arteriogenesis (in combination with angiogenesis) employed N^13^-ammonia (getting metabolically trapped in viable tissue) PET perfusion imaging, there are also studies that used SPECT imaging to link augmented myocardial perfusion to enhanced arteriogenesis.

Crottogini et al. investigated the effect of intramyocardial plasmid-mediated human VEGF_165_ gene transfer on the proliferation of vessels with smooth muscle in a pig model of myocardial ischemia. Using ^99m^Tc-sestamibi SPECT imaging, they reported enhanced myocardial perfusion accompanied by a significant increase in small sized collateral vessels compared to placebo treated pigs. However, angiographic quantification of collateral development using the Rentrop score failed to indicate a significant difference between the groups [156]. In a different study by the same group, Janavel et al. investigated the effect of VEGF gene transfer on the evolution of experimental myocardial infarction in adult sheep. They found an increase in angiogenesis (7 days after coronary artery ligation) and arteriogenesis (10 and 15 days after coronary artery ligation). Additionally, using ^99m^Tc-sestamibi SPECT, they found increased resting myocardial perfusion in VEGF-treated sheep 15 days after coronary artery ligation [157].

Although several studies relate enhanced perfusion and smooth muscle positivity to increased arteriogenesis, caution has to be taken when using this ambiguous term. Both the maturation of angiogenic vessels as well as collateral formation are coined arteriogenesis despite the difference in impact they have on perfusion recovery. While both cause enhanced smooth muscle detectability, collateral formation is more potent to drive perfusion. Hence, ascribing perfusion recovery to collateral formation requires more than showing an increase in smooth muscle positivity. Validation of nuclear perfusion imaging with angiography (using the Rentrop scoring index) will likely provide a better insight into whether perfusion recovery is caused by maturation of angiogenic vessel or collateral formation. For example, in a Yorkshire swine ameroid contrictor model, Mack et al. linked significant ^99m^Tc-sestamibi SPECT perfusion recovery in VEGF_121_ adenovector injected animals to significantly enhanced Rentrop scores (ex vivo coronary angiography) compared to control animals. However, no αSMA staining was performed [158]. In the same pig myocardial ischemia model, several other studies reported enhanced collateralization and perfusion after therapeutic stimulation, although no nuclear perfusion imaging was performed. For example, in a pig ameroid contrictor model, Tio et al. showed enhanced myocardial perfusion (indicated by colored microspheres), in animals intramyocardially injected with a VEGF_165_ -encoding DNA containing plasmid. Enhanced perfusion at maximal vasodilation (adenosine) was accompanied by an increased Rentrop score compared to control animals [159]. In a similar model, Sato et al. investigated the effect of intracoronary administration of FGF2. Using angiography, they found significant improvement in collateralization (assessed by Rentrop scoring), which was supported by enhanced perfusion (microspheres and MRI) and function (MRI) [160]. More evidence on the collateralization enhancing effects of FGF2 was gathered by Laham et al., also in a pig model of myocardial ischemia. Using angiography, they found enhanced collateralization (Rentrop scoring). Additionally, improved myocardial perfusion (microspheres and MRI) and function (MRI) in the ischemic territory were found. Moreover, histological evidence of increased myocardial vascularity was reported [161].

### Radiotracer imaging of arteriogenesis in models of peripheral vascular disease

Stacy et al. investigated serial changes in lower extremity arteriogenesis and muscle perfusion in a pig model for PVD [162]. Significant increases in collateral artery formation in the biceps femoris and semimembranosus muscle area were shown using CT angiography 4 weeks after ligation. They concluded that arteriogenesis within the semimembranosus and biceps femoris presumably resulted in improved downstream perfusion, which was quantified using Tl^201^ SPECT and validated by postmortem gamma-counting 4 weeks after induction of hindlimb ischemia.

More recently, our own group used ^99m^Tc-sestamibi in a mouse model for PVD to show that perfusion recovery through arteriogenesis after femoral artery ligation appears to happen much faster than suggested by standard laser-Doppler perfusion imaging. Perfusion in the ligated hind limb restored to levels comparable to the control limb on day 7 after ligation surgery and was accompanied by a significant increase in collateral artery diameter (αSMA). Additionally, ^99m^Tc-pyrophosphate was used to indicate muscular damage. Peak uptake of ^99m^Tc-pyrophosphate was found 3 days after femoral artery ligation, which recovered to baseline levels 14 days after surgery. The ^99m^Tc-pyrophosphate data was further invigorated by histological findings showing peak monocyte/macrophage infiltration (CD68 staining) and DNA fragmentation (TUNEL staining) on day 3 post femoral artery ligation [163].

## Developing candidate tracers for arteriogenesis imaging

Molecular radiotracers for imaging the arteriogenic process are lacking despite the fact that there is no scarcity in theoretic targets. Due to the larger diameters of collaterals, classic, non-nuclear imaging (i.e. microspheres and angiography) has been used for detection and quantification. However, these methods are restricted to late stage arteriogenesis and lack sensitivity or the quantitative capacity compared to nuclear imaging techniques such as SPECT or PET. Early detection of arteriogenesis through radiotracer imaging might be a valuable diagnostic with therapeutic or prognostic implications. As growing collateral arteries are hallmarked by the upregulation of adhesion molecules and subsequent invasion of monocytes (followed by T-lymphocytes) which in turn are a rich source of different cytokines [164], there is a variety of mechanisms that can be targeted through radiotracer imaging. Although there is definitely an inflammatory component involved in the arteriogenic process [165, 166], for example the MCP-1 pathway that recruits monocytes to areas of collateral artery development [167], applying ligands that bind to inflammatory targets might not be suitable for operating in a transient inflammatory environment.

Designing tracers for arteriogenesis imaging requires suitable targets that have the potential to be labeled with a radiotracer (e.g. through attachment of a chelating agent). Furthermore, a meaningful animal model both for cardiac arteriogenesis as well as arteriogenesis in PVD, and a sensitive and specific imaging readout are needed. To study arteriogenesis in the setting of PVD, we have been using a mouse [163] and rat [149] hindlimb model that are well established models of collateral formation. For the cardiac arteriogenesis, we have been using a mouse infarct model [168, 169], although the distinction between angiogenesis and arteriogenesis cannot be clearly made. Furthermore, a porcine model of chronic myocardial ischemia by an ameroid contrictor has been used in combination with whole mount cryomicrotome imaging to unambiguously show collateral development [165].

Targets we have been chasing include an ICAM-1 antibody, chemokines such as CXCL1, and other ligands that bind to chemokines (e.g. Evasin3, binding to CXCL1). When designing tracers, we have focused on a double-labeling approach (i.e. fluorescent tag, as well as the ability to attach a radioisotope through a chelator). We opted for this approach to enable correct spatiotemporal presentation of target binding of our tracers. While the fluorescent tag facilitates the option to perform fluorescence microscopy or high resolution two-photon laser scanning microscopy (TPLSM), the chelator provides the option for radiotracer imaging. Subsequent chasing experiments with non-radiolabeled agents could serve as indication for target binding specificity.

So far, these novel tracers, however, proved to be ineffective as imaging agents. Reasons for this failure might be that most targets reside at endothelial cells, are expressed at low intensity and exposed to a high flow circulatory environment. Nevertheless, while the target should be abundantly expressed in the area of collateralization, it should not (or only marginally) be expressed in non-specific areas. The monoclonal anti ICAM antibody we tested in vivo appeared to bind to constitutively expressed ICAM-1 on the endothelial lining in every vessel, thereby making it impossible to distinguish specific binding patterns. Moreover, it is conceivable that an arteriogenesis specific tracer should engage in polyvalent binding before being able to overcome the high shear stress in the area of collateralization.

## Conclusion

Advances in radiotracer imaging are made through continuously improving camera hard- and software as well as through the development of new radiotracers with favorable imaging characteristics. In this review, we discussed the role of perfusion tracers and provided an extensive overview of pre-clinical research into radiotracer imaging of angiogenesis and arteriogenesis in the context of MI and PVD.

Abundant pre-clinical research has resulted in the identification and development of angiogenesis tracers, while the development of specific arteriogenesis tracers remains largely overlooked. So far, perfusion tracers have been used to indicate enhanced perfusion through arteriogenesis (in combination with angiogenesis). Currently, only a few angiogenesis (targeting the α_v_β_3_ integrin) tracers have found their way to the clinic and broad-scale implementation of specific angiogenesis and arteriogenesis imaging in MI and PVD patients is still lacking. Future research should therefore focus on improving the translation of neovascularization tracers into the clinic, especially in the case of arteriogenesis. However, as illustrated in the last section, designing a tracer, specific for arteriogenesis imaging is not easy. When designing an arteriogenesis tracer, it is important to keep in mind the high shear stress environment that has to be overcome (potentially requiring polyvalent binding), as well as the notion that inflammatory factors might not operate well within a transient inflammatory setting. Clinical implementation of specific angiogenesis and arteriogenesis imaging can aid tailored therapy and would be a huge asset to MI and PVD patient risk stratification.
